# Supplementary material for: Constrained shadow tomography for molecular simulation on quantum devices
Source: Chem Sci. 2026 Mar 27;17(19):9633–43. doi: 10.1039/d5sc09162d (PMC13023410; doi:10.1039/d5sc09162d)
Supplement: SC-017-D5SC09162D-s001 [file SC-017-D5SC09162D-s001.pdf]

Supplementary Information (SI) for Chemical Science.
This journal is © The Royal Society of Chemistry 2026

Supplemental Material: Constrained Shadow Tomography for Molecular Simulation on Quantum Devices

Irma Avdic,[1] Yuchen Wang,[1,*] Michael Rose,[1,*] Lillian I. Payne Torres,[1] Anna O. Schouten,[1] Kevin J. Sung,[2,†] and David A. Mazziotti[1,‡]

[1]*Department of Chemistry and The James Franck Institute, The University of Chicago, Chicago, IL 60637 USA*
[2]*IBM Quantum, IBM T.J. Watson Research Center, Yorktown Heights, NY 10598 USA*

(Dated: Submitted November 13, 2025; Revised February 21, 2026)

CONTENTS

I. NUMERICAL SIMULATION DETAILS 1
A. Shadow Variational Two-Particle Reduced Density Matrix (sv2RDM) Method 1
B. Fermionic Classical Shadows (FCS) 2

II. QUANTUM HARDWARE DETAILS 3
A. H_4 Rectangle-Square-Rectangle Transition 3
B. Linear Hydrogen Chains 3
C. 2-RDM Reconstruction and Post-Processing 4

III. ADDITIONAL DATA 4

References 6

I. NUMERICAL SIMULATION DETAILS

A. Shadow Variational Two-Particle Reduced Density Matrix (sv2RDM) Method

To simulate measurement uncertainty, we generate noisy two-particle reduced density matrices (2-RDMs) by adding element-wise Gaussian perturbations to the full configuration interaction (FCI) 2-RDMs, where each noise term models the statistical fluctuations arising from finite-shot quantum measurements. Specifically, the variance of the noise for each matrix element is taken as

$$\sigma = \sqrt{\frac{p(1-p)}{N_{shots}}}. \tag{1}$$

This expression follows from the binomial variance of repeated independent measurements (Bernoulli trials), where the success probability p reflects the likelihood of finding electrons in particular spin orbitals. In quantum systems, such probabilities originate from the one-particle reduced density matrix (1-RDM), whose eigenvalues (natural orbital occupations) encode the marginal probabilities of electron occupation in each orbital. In Slater determinants (mean-field wavefunctions), these occupations are exactly 0 or 1. However, in correlated many-electron states, the occupations lie in the interval [0,1], introducing measurement uncertainty when estimating these probabilities from finite samples. Repeated projective measurements of such orbitals yield sample frequencies with binomial statistics, which, in the large-shot limit, converge to Gaussian distributions under the Central Limit Theorem. By modeling each matrix element of the 2-RDM as an effective outcome derived from such orbital occupations, we apply Gaussian-distributed noise consistent with these binomial variances.

For each measurement setting i, the spin-free 1- and 2-RDMs were obtained as described above and subsequently spin-adapted to yield the singlet and triplet components. Corresponding antisymmetric and symmetric unitary

* These authors contributed equally to this work.
† kevinsung@ibm.com
‡ damazz@uchicago.edu

transformations were used to generate transformed operator bases. To generate a random unitary matrix, we first sample the entries from a standard normal distribution with a mean of 0 and variance of 1. We then use the Gram-Schmidt decomposition to orthonormalize the columns of this random matrix. In random matrix theory, a matrix with independent identically distributed Gaussian entries whose columns have been orthonormalized via the Gram-Schmidt process is known to be Haar-distributed [1–3]. For each setting, linear constraints were accumulated across all unitaries to define the global feasible shadow constraint set. The correlated 2-RDM was obtained by solving the semidefinite program given in Eq. 9 in the manuscript with $w = 1$, using the Maple Quantum Chemistry Package. The recovered energy was computed as

$$E = E_{\mathrm{nuc}} + E_{\mathrm{elec}} - \sum_i w_i \mathrm{Tr}(\mathbf{E}_i), \tag{2}$$

where E_{nuc} is the nuclear energy, E_{elec} is the electronic energy, $\mathbf{E}_i$ is a positive semidefinite slack error matrix, and w_i is a regularization parameter introduced in Eq. 8 in the manuscript. Final energies were averaged across all iterations.

B. Fermionic Classical Shadows (FCS)

We briefly outline the derivation of the particle number-conserving Fermionic Classical Shadows (FCS) protocol as presented in Ref. [4].

A single-particle basis rotation acting on the η-particle sector of Fock space is defined as

$$U_\eta(u) \doteq e^{\sum_{p,q=1}^{n} [\ln(u)]_{pq} a_p^\dagger a_q}, \tag{3}$$

where $u \in \mathbb{C}^{n\times n}$ is Haar-random. Measuring in this rotated basis yields projectors $\Pi_z = |\vec{z}\rangle\langle\vec{z}|$ sampled with probability $\langle\psi|U_\eta^\dagger(u)\Pi_z U_\eta(u)|\psi\rangle$. The corresponding shadow estimator is

$$\hat{\rho}_{u,\vec{z}} \doteq \mathcal{M}_{\mathcal{U}}^{-1}\left[U_\eta^\dagger(u)|\vec{z}\rangle\langle\vec{z}|U_\eta(u)\right]. \tag{4}$$

For each occupation vector $|\vec{z}\rangle$, we introduce a permutation unitary $U(v_z)$ such that $U(v_z)|[\eta]\rangle = |\vec{z}\rangle$, where $|[\eta]\rangle$ is a vector representing the occupation vector in the permuted basis, allowing us to express

$$\hat{\rho}_{u,\vec{z}} = \mathcal{M}_{\mathcal{U}}^{-1}\left[U_\eta^\dagger(u)U_\eta(v_z)|[\eta]\rangle\langle[\eta]|U_\eta^\dagger(v_z)U_\eta(u)\right]. \tag{5}$$

Using the homomorphism property of fermionic rotations, $U_\eta^\dagger(u)U_\eta(v_z) = U_\eta^\dagger(v_z^\dagger u)$, this simplifies to

$$\hat{\rho}_{u,\vec{z}} = U_\eta^\dagger(v_z^\dagger u)\, \mathcal{M}_{\mathcal{U}}^{-1}[|[\eta]\rangle\langle[\eta]|]\, U_\eta(v_z^\dagger u). \tag{6}$$

To obtain reduced quantities, consider the k-body reduced density matrix (k-RDM)

$$D = \sum_{\vec{p},\vec{q}\in\mathcal{S}_{n,k}} d_{\vec{p},\vec{q}} D_{\vec{q}}^{\vec{p}}, \quad D_{\vec{q}}^{\vec{p}} \doteq a_{p_1}^\dagger \cdots a_{p_k}^\dagger a_{q_k} \cdots a_{q_1}. \tag{7}$$

Expectation values can then be evaluated as

$$\langle D_{\vec{q}}^{\vec{p}}\rangle = \mathrm{Tr}\Big(D_{\vec{q}}^{\vec{p}} U_\eta^\dagger(v_z^\dagger u)\mathcal{M}_{\mathcal{U}}^{-1}[|[\eta]\rangle\langle[\eta]|]\, U_\eta(v_z^\dagger u)\Big), \tag{8}$$

which can be efficiently expressed in the k-particle subspace as

$$\langle \hat{D}_{\vec{q}}^{\vec{p}}\rangle = \langle\vec{q}|U_k^\dagger(v_z^\dagger u)E_{\eta,k}U_k(v_z^\dagger u)|\vec{p}\rangle, \tag{9}$$

where

$$E_{\eta,k} = \sum_{\vec{r}\in\mathcal{S}_{n,k}} |\vec{r}\rangle\langle\vec{r}|\, \frac{\binom{\eta-s'}{k-s'}\binom{n-\eta+s'}{s'}}{(-1)^{k+s'}\binom{k}{s'}}, \qquad s' = |\vec{r}\cap[\eta]|, \tag{10}$$

with average variance

$$\mathcal{V} \leq \binom{\eta}{k}\left(1-\frac{\eta-k}{n}\right)^{k}\frac{1+n}{1+n-k}. \tag{11}$$

Each configuration $|\vec{r}\rangle$ corresponds to a term in the k-RDM, providing a compact and computationally tractable formulation of fermionic shadow estimation.

In practice, the unitary $U_\eta(u)$ defined in Eq. 3 cannot be evaluated explicitly for large systems on a classical computer, as it acts on a Hilbert space of dimension $\binom{n}{\eta}$, which scales exponentially with η. Instead, we compute the generator

$$K = \sum_{p,q=1}^{n} [\ln(u)]_{pq} a_p^\dagger a_q, \tag{12}$$

and implement the transformation via a Pauli-sum evolution $e^K|\psi\rangle$. This approach enables the efficient evaluation of $U_\eta(u)$ within variational or simulation frameworks such as Jordan–Wigner representations.

For a fixed, small k, $U_k(u)$ is of manageable size, allowing classical evaluation. From Eq. (20) of Ref. [4],

$$U_\eta(u)|\vec{z}\rangle = \bigwedge_{k=1}^{\eta} |u_{z_k}\rangle = \sum_{\vec{p}\in\mathcal{S}_{n,\eta}} \det[u_{\vec{p}\vec{z}}]\,|\vec{p}\rangle, \tag{13}$$

which leads to

$$[U_k(u)]_{\vec{p},\vec{q}} = \det(u_{\vec{p},\vec{q}})\,, \tag{14}$$

where $u_{\vec{p},\vec{q}}$ denotes the submatrix of u formed by the corresponding indices. The permutation matrix $U(v_z)$ satisfying $U(v_z)|[\eta]\rangle = |\vec{z}\rangle$ can be computed classically. In the single-particle case (1-spin-orbital basis), this reduces to $U(v_z) = v_z$. The matrix product $v_z^\dagger u$ is computed directly, forming the argument for subsequent U_η and U_k constructions. The operator

$$E_{\eta,k} \doteq \sum_{\vec{r}\in\mathcal{S}_{n,k}} |\vec{r}\rangle\langle\vec{r}|\,\frac{\binom{\eta-s'}{k-s'}\binom{n-\eta+s'}{s'}}{(-1)^{k+s'}\binom{k}{s'}}, \qquad s' = |\vec{r}\cap[\eta]|\,, \tag{15}$$

is diagonal in the computational basis and can be evaluated efficiently using classical post-processing.

II. QUANTUM HARDWARE DETAILS

All quantum circuits used for data collection were implemented using the Qiskit [5] package and the ffsim [6] library on IBM's 156-qubit superconducting processor ibm_fez.

A. H_4 Rectangle-Square-Rectangle Transition

The data collection details here correspond to Fig. 5 in the manuscript. The compiled circuits consisted primarily of single-qubit $R_Z(\theta)$ and S_X rotations, interleaved with two-qubit controlled-Z entangling gates arranged according to the molecular connectivity pattern. The action was confined to nine device qubits, $(q_{123}, q_{124}, q_{136}, q_{143}, q_{144}, q_{145}, q_{146}, q_{147}, q_{148})$, with projective measurements on eight of these (q_{144} idle at readout). The effective circuit depth was $d_{\mathrm{DAG}} \approx 90$ layers. 20 iterations of 16 different unitary bases, each with 10,000 shots, were used.

B. Linear Hydrogen Chains

The data collection details here correspond to Table II in the manuscript. The compiled circuits consisted primarily of single-qubit $R_Z(\theta)$ and S_X rotations, interleaved with two-qubit controlled-Z entangling gates. For H_4, the action was confined to nine device qubits, $(q_{123}, q_{124}, q_{136}, q_{137}, q_{143}, q_{144}, q_{145}, q_{146}, q_{147})$, with projective measurements on

eight of these (q_{144} idle at readout). The effective circuit depth was $d_{\mathrm{DAG}} \approx 100$ layers. 16 different unitary bases, each with 10,000 shots, were used.

For H_6, the action was confined to 14 device qubits, ($q_{122}, q_{123}, q_{124}, q_{125}, q_{126}, q_{127}, q_{136}, q_{137}, q_{142}, q_{143}, q_{144}, q_{145}, q_{146}, q_{147}$), with projective measurements on 12 of these (q_{136}, q_{137} idle at readout). The effective circuit depth was $d_{\mathrm{DAG}} \approx 160$ layers. 36 different unitary bases, each with 10,000 shots, were used.

For H_8, the action was confined to 18 device qubits, ($q_{120}, q_{121}, q_{122}, q_{123}, q_{124}, q_{125}, q_{126}, q_{127}, q_{136}, q_{137}, q_{140}, q_{141}, q_{142}, q_{143}, q_{144}, q_{145}, q_{146}, q_{147}$), with projective measurements on 16 of these (q_{136}, q_{137} idle at readout). The effective circuit depth was $d_{\mathrm{DAG}} \approx 200$ layers. 160 different unitary bases, each with 10,000 shots, were used.

For H_{10}, the action was confined to 23 device qubits, ($q_{97}, q_{105}, q_{106}, q_{107}, q_{108}, q_{109}, q_{110}, q_{117}, q_{118}, q_{123}, q_{124}, q_{125}, q_{126}, q_{127}, q_{128}, q_{129}, q_{136}, q_{137}, q_{143}, q_{144}, q_{145}, q_{146}, q_{147}$), with projective measurements on 20 of these ($q_{108}, q_{126}, q_{144}$ idle at readout). The effective circuit depth was $d_{\mathrm{DAG}} \approx 250$ layers. 300 different unitary bases, each with 10,000 shots, were used.

C. 2-RDM Reconstruction and Post-Processing

Upon measurement, the obtained bitstrings were post-processed to reconstruct RDMs used in the subsequent semidefinite programming (SDP) procedure. Each measurement outcome was represented as a 64-bit integer, which was unpacked into a binary vector $\mathbf{b} \in \{0,1\}^{2n_{\mathrm{spatial}}}$ corresponding to occupations of the spin orbitals $\{i\alpha, i\beta\}$. Spin occupations were then summed to obtain spatial occupations. From these data, the diagonal elements of the spatial 1- and 2-RDMs were computed as statistical averages over all measurement shots. The diagonal elements of the 1-RDM were given by

$$ {}^1D^i_i = \langle n_i \rangle, \tag{16} $$

representing the mean occupation of each spatial orbital. The diagonal block of the spatial 2-RDM was obtained directly from pairwise orbital co-occupations,

$$ {}^2D^{ij}_{ij} = \langle n_i n_j \rangle, \tag{17} $$

which captures the joint probability that orbitals i and j are simultaneously occupied across all measured bitstrings. These averaged RDM elements were then assembled into matrix and tensor forms and expressed in Maple format for compatibility with subsequent SDP reconstruction and energy evaluation routines in Maple's Quantum Chemistry Package [7, 8].

Each random unitary basis for each point along the potential energy curve was subsequently validated by comparing its corresponding reconstructed 2-RDM against the FCI reference 2-RDM. While FCI was used as the reference in this instance, any method capable of providing a reliable 2-RDM may be used in its place. The deviation was quantified using the Frobenius norm,

$$ \Delta_2 = \left\| {}^2D_{\mathrm{unitary}} - {}^2D_{\mathrm{FCI}} \right\|_2, \tag{18} $$

which provides a global measure of the 2-RDM discrepancy. Unitaries yielding $\Delta_2 > 1$ were deemed inconsistent with the target electronic structure and were discarded from further analysis. Along the potential energy curve of H_4 (Fig. 5 in the manuscript) ranging from 0.65 to 1.65 Å, the following numbers of unitary bases (out of 16 for each point) were discarded at each geometry: 2, 2, 5, 6, 6, 5, 7, 5, 4, 2, and 3, respectively. For the data on linear hydrogen chains (H_4, H_6, H_8, H_{10}), the following numbers of unitary bases (out of 16, 36, 160, and 300, respectively), were discarded: 3, 8, 19, 37.

III. ADDITIONAL DATA

Figure S1 supplements Fig. 4 in the manuscript, presenting the simulated potential energy curve of N_2 in the cc-pVDZ basis set with a [10,8] active space using complete active space configuration interaction (CASCI), shadow variational 2-RDM (sv2RDM), variational 2-RDM (v2RDM), and fermionic classical shadows (FCS) methods. The CASCI results serve as the reference for evaluating the accuracy of the reconstructed energy profiles. The v2RDM energies are significantly lower than the CACSI reference, by as much as 20 milihartrees at longer distances. In contrast, the sv2RDM method closely reproduces the reference curve across all geometries, demonstrating the constrained shadow approach effectively mitigates the deviations observed with v2RDM.

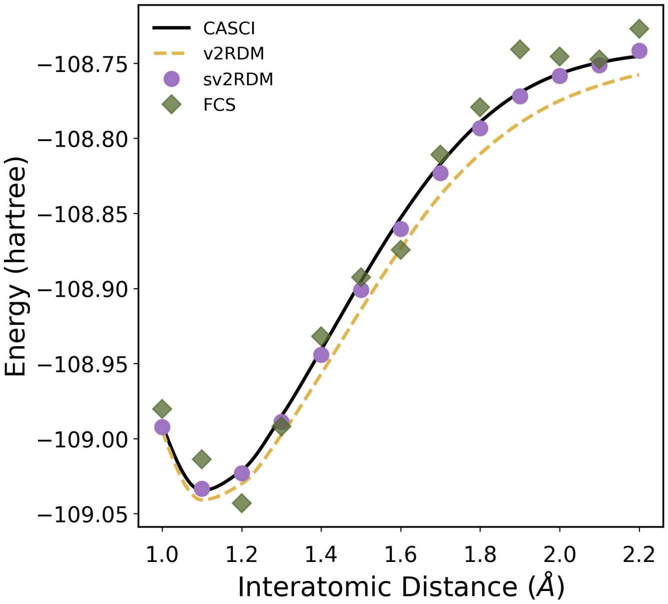

FIG. S1. Potential energy curve of N_2 computed using the cc-pVDZ basis set and a [10,8] active space with CASCI, shadow variational 2-RDM (sv2RDM), variational 2-RDM (v2RDM), and Fermionic Classical Shadows (FCS) methods. For sv2RDM and FCS, the total shot budgets used are 100 unitaries × 1,000 shots and 100,000 unitaries × 1 shot, respectively.

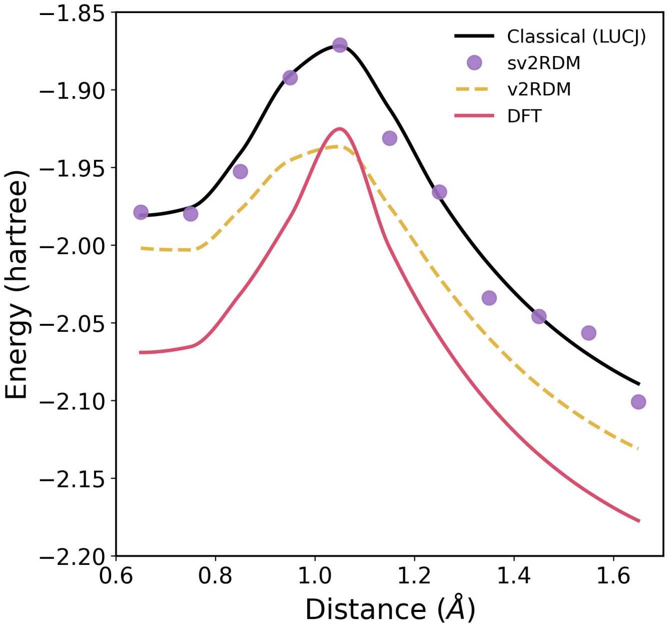

FIG. S2. Potential energy curve for the H_4 rectangle–square–rectangle transition in the minimal basis set, computed using Density Functional Theory (DFT) as s classical baseline, the classically computed Local Unitary Coupled Cluster with Jastrow (LUCJ) ansatz, shadow variational 2-RDM (sv2RDM), and variational 2-RDM (v2RDM) method computed using measurements collected on the ibm_fez quantum computer.

Figure S2 supplements Fig. 5 in the manuscript, showing the potential energy curve for the H_4 rectangle–square–rectangle transition in the minimal basis set, computed using Density Functional Theory (DFT) as a classical baseline, the classically computed Local Unitary Coupled Cluster with Jastrow (LUCJ) ansatz, variational 2-RDM (v2RDM), and the shadow variational 2-RDM (sv2RDM) methods, where sv2RDM is computed using measurements collected on the ibm_fez quantum computer. The v2RDM curve qualitatively reproduces the expected features of the transition

H chain	Shot Budget	D	DQG	FCI
4	16,000	-2.122	-2.121	-2.166
6	36,000	-3.129	-3.122	-3.236
8	160,000	-4.152	-4.142	-4.308
10	300,000	-5.161	-5.144	-5.380

TABLE S1. Comparison of electronic energies (in hartree) for various H chain lengths with the given total shot budgets when the semidefinite 2-RDM is optimized in the limit where $w \to \infty$ in Eq. 11 in the main text, with partial (D) and all 2-positivity conditions (DQG) included. Shot budgets compared are: 16,000 unitaries/1 shot vs. 16 unitaries/1000 shots for H_4, 36,000 unitaries/1 shot vs. 36 unitaries/1000 shots for H_6, 160,000 unitaries/1 shot vs. 160 unitaries/1000 shots for H_8, and 300,000 unitaries/1 shot vs. 300 unitaries/1000 shots for H_{10}.

and aligns more closely with the correlated LUCJ profile than the mean-field DFT results. The sv2RDM results, computed using data obtained from a quantum computer, exhibit very close agreement with the LUCJ curve across the full range of geometries, indicating that the constrained shadow reconstruction yields a quantitatively accurate potential energy curve using data collected on a quantum computer.

To illustrate the energetic performance of the reconstruction procedure in the fully state-agnostic regime, Table S1 reports equilibrium electronic energies for linear hydrogen chains obtained from SDP-optimized 2-RDMs in the limit $w \to \infty$, where no Hamiltonian-dependent energy regularization is included in the cost function. In this setting, the reconstruction is driven entirely by measurement data and N-representability constraints. As expected, energy expectation values are more sensitive to residual reconstruction errors, leading to deviations from the FCI reference that grow with system size. Nevertheless, this Hamiltonian-independent regime can be practically useful, as discussed in related work by the authors [9], the $w \to \infty$ limit provides a natural setting for correlated purification and error mitigation, and enables reconstruction beyond ground-state assumptions, including excited-state applications.

[1] Diaconis, P. Patterns in Eigenvalues: The 70th Josiah Willard Gibbs Lecture. *Bulletin of the American Mathematical Society* **2003**, *40*, 155–178, Article electronically published on February 12, 2003.

[2] Mezzadri, F. How to generate random matrices from the classical compact groups. *arXiv* **2006**,

[3] Maplesoft Generating random unitary matrices. `https://www.mapleprimes.com/questions/227248-Generating-Random-Unitary-Matrices`, n.d.; [Online forum post].

[4] Low, G. H. Classical Shadows of Fermions with Particle Number Symmetry. *arXiv* **2022**,

[5] Qiskit contributors Qiskit: An Open-source Framework for Quantum Computing. 2025.

[6] The ffsim developers ffsim: Faster simulations of fermionic quantum circuits. `https://github.com/qiskit-community/ffsim`.

[7] *Maple*; Maplesoft, Waterloo, 2025.

[8] *RDMChem, Quantum Chemistry Package*; Maplesoft, Waterloo, 2025.

[9] Wang, Y.; Avdic, I.; Rose, M.; Torres, L. I. P.; Schouten, A. O.; Sung, K. J.; Mazziotti, D. A. Correlated Purification for Restoring N-Representability in Quantum Simulation. 2025; `https://arxiv.org/abs/2511.10789`.
